# VLA4-Enhanced Allogeneic Endothelial Progenitor Cell-Based Therapy Preserves the Aortic Valve Function in a Mouse Model of Dyslipidemia and Diabetes

**DOI:** 10.3390/pharmaceutics14051077

**Published:** 2022-05-17

**Authors:** Alexandru Filippi, Alina Constantin, Nicoleta Alexandru, Cristina Ana Mocanu, Mihaela Loredana Vlad, Ioana Madalina Fenyo, Agneta Simionescu, Dan Teodor Simionescu, Ileana Manduteanu, Adriana Georgescu

**Affiliations:** 1 Department of Pathophysiology and Pharmacology, Institute of Cellular Biology and Pathology “Nicolae Simionescu” of the Romanian Academy, 050568 Bucharest, Romania; alexandru.filippi@umfcd.ro (A.F.); alina.constantin@icbp.ro (A.C.); nicoleta.alexandru@icbp.ro (N.A.); 2 Department of Biophysics, University of Medicine and Pharmacy “Carol Davila”, 050474 Bucharest, Romania; 3 Department of Biopathology and Therapy of Inflammation, Institute of Cellular Biology and Pathology “Nicolae Simionescu” of the Romanian Academy, 050568 Bucharest, Romania; cristina.mocanu@icbp.ro (C.A.M.); ileana.manduteanu@icbp.ro (I.M.); 4 Laboratory of Molecular and Cellular Pharmacology-Functional Genomics, Institute of Cellular Biology and Pathology “Nicolae Simionescu” of the Romanian Academy, 050568 Bucharest, Romania; loredana.vlad@icbp.ro; 5 Laboratory of Gene Regulation and Molecular Therapies, Institute of Cellular Biology and Pathology “Nicolae Simionescu” of the Romanian Academy, 050568 Bucharest, Romania; madalina.fenyo@icbp.ro; 6 Department of Bioengineering, Clemson University, Clemson, SC 29634-0905, USA; agneta@clemson.edu (A.S.); dsimion@clemson.edu (D.T.S.)

**Keywords:** endothelial progenitor cells, integrin α4β1 (VLA4), aortic valve, dyslipidemia, diabetes

## Abstract

The number and function of endothelial progenitor cells (EPCs) are reduced in diabetes, contributing to deteriorated vascular repair and the occurrence of cardiovascular complications. Here, we present the results of treating early diabetic dyslipidemic mice or dyslipidemic with disease-matched EPCs modified to overexpress VLA4 (VLA4-EPCs) as compared with the treatment of EPCs transfected with GFP (GFP-EPCs) as well as EPCs from healthy animals. Organ imaging of injected PKH26-stained cells showed little pulmonary first-pass effects and distribution in highly vascularized organs, with splenic removal from circulation, mostly in non-diabetic animals. Plasma measurements showed pronounced dyslipidemia in all animals and glycaemia indicative of diabetes in streptozotocin-injected animals. Echocardiographic measurements performed 3 days after the treatment showed significantly improved aortic valve function in animals treated with VLA4-overexpressing EPCs compared with GFP-EPCs, and similar results in the groups treated with healthy EPCs and VLA4-EPCs. Immunohistochemical analyses revealed active inflammation and remodelling in all groups but different profiles, with higher MMP9 and lower P-selectin levels in GFP-EPCs, treated animals. In conclusion, our experiments show that genetically modified allogeneic EPCs might be a safe treatment option, with bioavailability in the desired target compartments and the ability to preserve aortic valve function in dyslipidemia and diabetes.

## 1. Introduction

Calcific aortic valve disease (CAVD) is a disorder that evolves progressively from mild valve thickening without the obstruction of blood flow to severe calcification leading to aortic valve stenosis (AVS). Elevated circulating levels of oxidized low-density lipoprotein (oxLDL) were correlated with aortic valve calcification, fibrosis and oxLDL accumulation in calcified and stenotic aortic valves were well described [1]. Calcific aortic valve stenosis (CAVS) is known as active disease, such as atherosclerosis, having similar risk factors, namely older age, male sex, hypertension, smoking, hypercholesterolemia, and diabetes; however, about 50% of patients with CAVS do not have clinically significant atherosclerosis [2,3]. Although diabetes has been closely associated with coronary atherosclerosis, its involvement in CAVS was later accepted. It is now known that diabetes can exacerbate the inflammatory effect and lipid accumulation in the calcified aortic valve, thus accentuating the CAVS progression [4]. An important causative role of diabetes, dyslipidaemia and hypertension in the pathogenesis and development of CAVS has been confirmed in the Cardiovascular Health in Ambulatory Care Research Team (CANHEART) study [5].

The development of new therapies for AVS has been impeded by a limited understanding of the complex mechanisms driving CAVD initiation and progression towards clinically relevant interventions. One of the first events triggering CAVD is the damage of the endothelium on both sides of the valve leaflets, resulting from local alterations of blood shear stress [6]. It has recently been shown that the valvular endothelial cells (VECs) and valvular interstitial cells (VICs) cultured in a gelatin methacrylate 3D construct and exposed to high glucose conditions presented increased expressions of various cytokines, cell adhesion molecules and matrix metalloproteinases, with a rapid and strong response in VECs, while in VICs the chronic inflammation developed and persisted longer [7]. Increased expressions of cell adhesion molecules (including Vascular Cell Adhesion Molecule-1 (VCAM-1)), extracellular matrix remodelling and osteogenic markers were detected in the aortic valve of hyperlipidemic ApoE^−/−^ diabetic mice [8].

To date, drug therapies used for lowering cholesterol circulatory levels [9] and renin-angiotensin system inhibitors [10] were ineffective to delay the progression of severe AVS, and thus surgical and transcatheter aortic valve replacement with a mechanical or bioprosthetic valve represents the only available option to improve clinical outcomes and to increase survival.

Cell therapy has been evidenced as a promising therapeutic option for treating patients with cardiovascular diseases [11]. Tissue Engineering of Heart Valves (TEHV) has emerged as a valuable alternative for the treatment of valve disease, and in vitro TEHV uses different types of stem/progenitor cells that are expanded in culture, seeded on decellularized biological or synthetic scaffolds that may be conditioned in a bioreactor to ensure fast and competent “natural-like” matrix production before implantation [12,13]). The cell types used for valve engineering are VICs, VECs, mesenchymal stem cells (MSCs) or medicinal signalling cells (as they were renamed by Caplan in 2017) [14], bone marrow-derived mononuclear cells (BM-MNCs), fibroblasts and endothelial progenitor cells (EPCs) [15].

With regard to the EPCs, it is well known that in diabetes their levels are significantly reduced [16,17] were negatively associated with glycated haemoglobin (HbA1c) levels [18]. The capacity of EPCs to differentiate into endothelial cells (ECs) and contribute to endothelial repair and vasculogenesis has already been demonstrated. When ECs are damaged, EPCs may replace them to aid in the recovery of endothelial function affected by atherosclerosis [19] and diabetes [20]. EPCs, isolated from a subset of circulating CD34-positive MNCs, possessed the ability to differentiate into ECs when cultured in vitro or to be incorporated into newly formed vessels upon transplantation in animal models of ischemia [21] or atherosclerotic cardiovascular disease [22].

The efficiency of cell therapy to augment recovery depends on the sufficient recruitment of applied cells to the target tissues. To ensure effective endothelial capture, injected EPCs must express key beta 1 and beta 2 integrins that facilitate their binding to the endothelial counterparts. Our recent results showed a reduced level of integrin alpha 4 beta 1 (α4β1), also called VLA4 (very late antigen-4), in circulatory EPCs from diabetic animals [23], in agreement with previous data linking PKA-mediated phosphorylation of α4β1with bone marrow retention of EPCs in diabetes [24]. The integrin VLA4 and its ligand VCAM-1 are a well-characterized receptor-ligand pair involved in leukocyte rolling and firm adhesion to sites of injured ECs. As low VLA4 levels could further contribute to a decreased recruitment of EPCs to the activated endothelium, we set forth to assay whether ex vivo modification of EPCs from diseased animals to overexpress the α4 and β1 integrin components of VLA4 could restore part of their function and preserve the aortic valve function; for this purpose, we used the animal model we previously developed to assay aortic valve modifications in early diabetes associated with dyslipidemia [8], the same model we also used for the EPC phenotype assessments [23].

## 2. Materials and Methods

### 2.1. Animal Models

The animals in this study included apolipoprotein E knock-out (ApoE^−/−^) mice from the breeding colony of Taconic Biosciences, bred in our facility, as well as C57BL6 mice, in specific pathogen-free (SPF) conditions, kept under a 12 h light:12 h dark cycle, with food and water ad libitum. In the experiments, both male and female mice (in different sets of experiments) aged 3 to 4 months were used.

The experimental models used for dyslipidemic and diabetic dyslipidemic mice were previously described in detail [8]. Briefly, to obtain diabetic dyslipidemic animals (STZ groups), from disease-prone ApoE^−/−^ mice, streptozotocin (STZ) was injected at 55 mg/kg of body weight for 5 consecutive days to induce diabetes and next the diet was changed to atherogenic food (standard pellets were enriched in cholesterol (1%) and butter (15%)) for another 4 days. Dyslipidemic (but not diabetic) animals (CIT groups) were obtained with the same protocol with the exception of injecting citrate buffer (CIT) instead of STZ.

The experiments aiming to assess the effects of genetically modified EPC administration used 31 (16 diabetic dyslipidemic and 15 dyslipidemic) male ApoE^−/−^ and 16 male C57BL/6 mice as a source of diseased EPCs and healthy EPCs, respectively. The EPCs from diabetic dyslipidemic and dyslipidemic animals (called diseased EPCs) were further divided into two groups (transfected with α4 and β1 integrins or green fluorescent protein (GFP)) and injected in a total of 24 male ApoE^−/−^ (as detailed below in the = subsection).

For a different set of experiments, assessing the in vivo distribution of the EPCs, 12 diabetic dyslipidemic ApoE^−/−^ and 11 dyslipidemic ApoE^−/−^ mice were used for the isolation and purification of EPCs as will be described below. EPCs were transfected either with GFP or α4 and β1 integrins to yield CIT-EPC-GFP, STZ-EPC-GFP, CIT-EPC-VLA4, and STZ-EPC-VLA4. EPCs from 8 healthy C57BL6 mice were used without any genetic modification. After staining with PKH26 (SIGMA), the EPCs were injected into 10 ApoE^−/−^ diabetic and dyslipidemic and, 10 ApoE^−/−^ dyslipidemic mice, respectively. In total, 43 ApoE^−/−^ and 8 C57BL6 female mice were used in the in vivo EPC distribution assays.

All mice were sacrificed by deep anaesthesia with ketamine-xylazine (80 mg/10 mg/kg body weight), the thoracic and abdominal cavities were surgically opened and the total fraction of blood (0.7–1 mL) was aspired through ventricular punction. All remaining blood was removed by perfusion with phosphate-buffered saline (PBS) and the organs were collected for further processing, as further described herein.

A flowchart detailing the animal model establishment protocol can be found in Supplementary Figure S1.

### 2.2. EPC Sorting

The peripheral blood mononuclear cell (PBMC) fraction, obtained by density gradient centrifugation of whole blood in Histopaque-1077 (SIGMA) according to the protocol described by Georgescu et al., 2012 [25], was stained with anti-VEGFR2 (KDR or Flk-1) and CD34 antibodies coupled with allophycocyanin (APC) and AlexaFluor 488, respectively. EPCs, identified as VEGFR2+/CD34+ monocytes, were sorted using MoFlo Astrios (Beckman Coulter) cell sorter (ex: 488 nm, em: 513/26 nm for AlexaFluor 488 and ex: 640 nm, em: 671/30 nm for APC), collected in 50% FBS (fetal bovine serum) in PBS and washed in PBS.

### 2.3. Transfection of EPCs

EPCs from diabetic dyslipidemic and dyslipidemic mice (diseased EPCs) were transfected to overexpress α4 and β1 integrins using Viromer^®^ RED by OriGene Technologies (Rockville, MD, USA), kit: TT100302, Buffer: B08-190711-VT) for intracellular delivery of plasmid DNA containing the coding sequences for the two proteins. Briefly, the plasmids were diluted in Viromer-RED-buffer for a final concentration of 11 ng/µL each. Afterwards, Viromer solution was prepared as follows: Viromer-RED was diluted 1:25 with Viromer-RED-buffer and vortexed for 5 s. To form the Viromer/plasmid DNA-complex, 45 μL of the plasmid dilution was mixed with 5 μL of the Viromer solution, homogenized and incubated for 15 min at room temperature. Subsequently, 100 µL of the Viromer/plasmid DNA-complex (transfection mix) were added to 200 µL of cell suspension (1 × 10^4^ diseased EPCs) and the cells were used after 72 h of culturing at 37 °C, 5% CO_2_ with slow, end-over-end rotation to avoid cell adherence to the tube [26,27].

As a control, the same protocol was used to transfect 1 × 10^4^ diseased EPCs with a GFP coding plasmid. Depending on the source animal and transfected proteins, the following types of cells were obtained following transfection:

- EPCs from dyslipidemic animals, transfected with GFP (CIT-EPC-GFP);
- EPCs from diabetic dyslipidemic animals, transfected with GFP (STZ-EPC-GFP);
- EPCs from dyslipidemic animals, transfected with α4 and β1 integrins (CIT-EPC-VLA4);
- EPCs from diabetic dyslipidemic animals, transfected with α4 and β1 integrins (STZ-EPC-VLA4).

### 2.4. Animal Groups and Their Treatments

**Figure 1:**
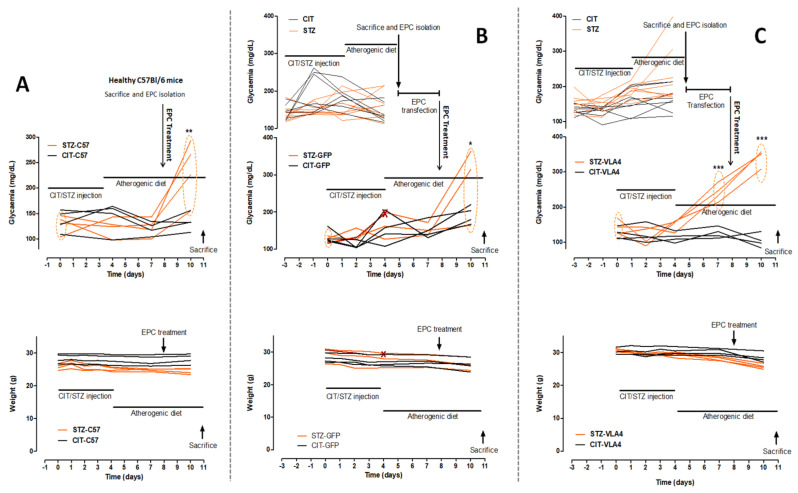
(**A**) Glycaemia (upper graph) and body weights (lower graph) of CIT/STZ mice treated with unmodified EPCs from healthy C57BL; (**B**) Glycaemia (upper graph) and body weights (lower graph) of CIT/STZ mice treated with GFP-transfected EPCs; (**C**) Glycaemia (upper graph) and body weights (lower graph) of CIT/STZ mice treated with EPCs transfected to overexpress α4 and β1 integrins. A moderate decrease in body weight can be observed for the diabetic dyslipidemic mice and one dyslipidemic mouse starting before the EPC injection, as induced by a more robust response to STZ, with significantly increased glycemic levels before EPC treatment. For the (**B**,**C**) experimental arms, the uppermost diagram represents the glycaemia of mice used to obtain EPCs for transfection. Traces from diabetic dyslipidemic animals (STZ mice) are shown in orange and those from dyslipidemic non-diabetic mice (CIT mice), are in black. * *p* < 0.05, ** *p* < 0.01, *** *p* < 0.001 compared with day 0 measurements.

The EPCs obtained after the transfection step were administered in a single dose by retro-orbital sinus injection containing 1 × 10^4^ EPCs/animal (ketamine-xylazine anaesthetized mice) to disease-matched animals (CIT group/STZ group) generating the following groups for therapy evaluation (see Figure 1 for the detailed timeline):

- CIT-EPC-GFP in dyslipidemic animals (n = 4 males and 2 females)
- STZ-EPC-GFP in diabetic dyslipidemic animals (n = 4 males and 2 females)
- CIT-EPC-VLA4 in dyslipidemic animals (n = 4 males and 4 females)
- STZ-EPC-VLA4 in diabetic dyslipidemic animals (n = 4 males and 4 females)

In addition, two other groups of animals were established to compare the organ distribution of diseased EPCs, transfected with α4 and β1 integrins or GFP, with the effect of unmodified EPCs from healthy C57BL6 mice (EPC-C57):

- CIT-EPC-C57 in dyslipidemic animals (n = 4 males and 2 females);
- STZ-EPC-C57 in diabetic dyslipidemic animals (n = 4 males and 2 females).

Moreover, CIT and STZ groups of animals not treated with EPCs were used as a control (autofluorescence):

- CIT-AFL-dyslipidemic animals (n = 2 females);
- STZ-AFL-diabetic dyslipidemic animals (n = 2 females).

While both male and female mice were used, they were involved in different experiments, the female mice being used only for the organ distribution assay, aiming to determine the EPC availability to target tissues, and not the specific effects said treatment might produce.

### 2.5. Biochemistry

Plasma was obtained by centrifuging at 2500 *g*, 4 °C for 10 min using the blood collected on ethylenediaminetetraacetic acid (EDTA) by ventricular punction; later, total cholesterol, HDL-cholesterol, LDL-cholesterol and triglyceride plasma concentrations were determined using colorimetric kits (Dialab GmbH, Wiener Neudorf, Austria), according to manufacturer instructions.

Plasma Fetuin A was measured using an ELISA kit (R&D Systems, Minneapolis, MN, USA). Glycated haemoglobin was measured from cell lysates by ELISA (Cusabio Biotech, Houston, TX, USA), using manufacturer instructions. For all biochemistry determinations, each sample was measured in duplicate, and the mean was used in further analysis; furthermore, glycaemia was measured using a commercial glucometer one week after STZ or vehicle administration, and later, every 2 days.

### 2.6. Ecocardiography

The aortic valve function was evaluated using an ultra-high frequency linear array transducer coupled to a Vevo2100 the High-Resolution Ultrasound Imaging System for small animals (FUJIFILM VisualSonics, Inc. (FF-VSI), Toronto, ON, Canada). Before the procedure, the hair on the ventral thorax of the animals was removed. During the procedure, the mice were kept under anaesthesia with 2% isoflurane, on a heated platform, under continuous monitoring of heart rate and body temperature. Echocardiographic data sets were acquired in standard parasternal long-axis view. Aortic valve blood velocity (VEL) and velocity-time integral (VTI) of the left ventricle outflow tract (LVOT) were recorded using the pulsed-wave Doppler mode (PW Doppler-mode); moreover, other recorded parameters were aortic leaflet width (in B-mode) and systolic aortic valve opening (in M-mode). All acquired images were digitally stored in raw format (DICOM) for further offline-analyses using the Vevo LAB software, version 3.0.0. (FF-VSI, Toronto, ON, Canada).

### 2.7. EPC Organ Distribution

To visualise and quantify PKH26-stained EPC distribution in organs, IVIS^®^ SpectrumCT In Vivo Imaging System (PerkinElmer, Waltham, MA, USA) was used. Because the black colour of ApoE^−/−^ mice derived from C57BL/6J would absorb most of the light used for excitation as well as emitted by PKH26, on whole animal scanning, EPC organ distribution was measured on isolated organs (lung, spleen, liver, kidney, heart, thoracic aorta) after animal sacrifice. To detect the specific fluorescence and correct for the nonspecific organ fluorescence (tissue autofluorescence), the acquisition was performed using two distinct wavelengths for excitation with four emission bands each and the autofluorescence was automatically subtracted by the acquisition software. The fluorescence results are recorded as absolute Radiant Efficiency, calculated using the formula:

Radiant Efficiency = (p/sec/cm^2^/sr)/(µW/cm^2^) where p is the number of photons, sec is the exposure time in seconds, cm^2^ is the measured area, sr is the angle of measurement in steradians and µW/cm^2^ is the excitation energy per square centimetre.

The results were quantified as mean fluorescence intensity ratio (MFIR), representing the mean fluorescence in the treatment group divided by the fluorescence measured in animals that were not injected with EPCs, as to indicate a fold increase in fluorescence in organs from treated animals.

### 2.8. Immunohistochemistry

Hearts were collected after the removal of all blood by ventricular punction and perfusion with PBS. To cryoprotect the tissues, the organs were immersed in increasing concentrations of glycerol (5% for 15 min, 10% for 1 h, 20% overnight and 50% until sectioning). After washing in 3% sucrose, the first apical two-thirds of the ventricles were incised and removed and the remaining sample was mounted in OCT (Neg-50; Thermo Fisher Scientific, Waltham, MA, USA) and snap-frozen in liquid nitrogen; 7 μm sections in the aortic valve plane were cut using a microtome (Leica CM1850), collected on microscopy slides and stored at −20 °C. For immunostaining, the aortic valve sections were incubated with primary antibodies (one each) in PBS plus 1% bovine serum albumin (BSA), for 2 h, at RT. The following primary antibodies were used: anti-P-selectin (mouse MA1-81809, 1:50; Thermo Fisher Scientific, Waltham, MA, USA), anti-metalloproteinase 9 (MMP9; rabbit PA5-27291, 1:500; Thermo Fisher Scientific) anti-fibronectin (mouse MA5-11981; Thermo Fisher Scientific), anti-smooth muscle alpha actin (αSMA; goat PA5-18292, 1:200; Santa Cruz) and bone morphogenetic protein 2 (BMP2; rabbit PA1-31215, 1:200; Thermo Fisher Scientific). After the primary antibody incubation, the sections were washed and incubated with secondary antibodies, coupled with fluorophores: donkey anti-mouse-AlexaFluor 594 (A322744; for P-selectin and fibronectin), donkey anti-goat-PE (PA1-29953, Thermo Fisher Scientific; for αSMA) and goat anti-rabbit-Rhodamine (sc-2091, SantaCruz; for MMP9 and BMP2), for 2 h, at RT. Nuclei were coloured with 4′,6-diamidino-2-phenylindole (DAPI) before mounting. The stained sections were visualised using a fluorescence microscope (Olympus IX81 equipped with an XC50 camera) and images of the valve were acquired at 40× (2–3 per valve section, to cover all leaflets present) with the same exposure and lamp intensity settings for all slides.

### 2.9. Processing and Data Analysis

For the fluorescence quantification from aortic valve microscopy images, the regions of interest (ROI) containing the valve leaflets were manually selected to exclude the signal from other tissues present in the image. For each ROI, the total fluorescence in the red channel (emission window of the fluorophores, chosen to minimise autofluorescence) and the total number of nuclei from the blue channel (DAPI staining) were analysed automatically using an ImageJ macro written for the task, yielding the ratio of total fluorescence/cell number.

Raw data obtained by echocardiography, biochemistry and organ imaging were organised in Excel and statistically analysed using GraphPad Prism; moreover, Python 3.8 was used for the calculation of Pearson coefficients (SciPy module), correlations matrix and remodelling profiles by radio diagrams visualisations (Matplotlib and Pandas).

## 3. Results

### 3.1. Generation of the Animal Model

In the cohorts used for EPC isolation, no body weight change was observed, indicative of incipient dyslipidaemia and diabetes, with no complications (data not shown). Mean glycaemia showed relatively stable levels, with a slight increase in STZ injected animals, at 7 days after the first dose (Figure 1, upper diagrams, orange tracks). The treatment of these animals with STZ or CIT and an atherogenic diet was started 3 days before that of the main experimental groups to accommodate the 3-day long EPC purification and transfection with α4 and β1 integrins (as well as GFP control) protocols. Following transfection, the mRNA levels of α4 integrin were increased about 9 times and those of β1 integrin was increased about 6 times compared to control, untransfected EPCs as measured by RT-PCR (Supplementary Figure S2).

EPCs thus obtained were injected in male ApoE^−/−^ mice, as detailed in Table 1.

To mimic an autologous transplant, the EPC treatment was performed on the same corresponding day of the disease progression as the EPC isolation and the animals were monitored for another 3 days before sacrifice and sample collection (see Figure 1 for the detailed timeline). Some of these animals showed a moderate decrease in body weight associated with the progression of the disease, more pronounced in diabetic dyslipidemic animals than in dyslipidemic animals. As expected, glycaemia was significantly increased in STZ-injected animals compared to CIT control for all treatment conditions (Figure 1A–C).

One mouse in the STZ-GFP group was found dead after the 4th STZ injection before EPCs could be administered, and thus, the results reported for this group were measured in 3 out of the initial 4 mice.

#### Biochemical Characterization

The experimental duration was long enough to induce significant increases in glycaemia in all STZ-injected groups (final measured glycaemia of 235 ± 60.02 mg/dL, *p* < 0.01 for STZ-EPC-C57, 280.7 ± 103.8 mg/dL, *p* < 0.05 for STZ-EPC-GFP and 342.5 ± 23.19 mg/dL, *p* < 0.001, for STZ-EPC-VLA4), with mice in the STZ-EPC-VLA4 group being even more responsive to diabetes induction (240.5 ± 24.42 mg/dL before EPC treatment, *p* < 0.001); however, the model studied being that of incipient diabetes, the time length was too short to observe significant increases in glycosylated haemoglobin (Table 2).

An atherogenic diet led to dyslipidemia in all treatment groups, with ample increases in total cholesterol and LDL cholesterol compared with the normal reference levels. Moreover, animals in the STZ-EPC-GFP group showed a significantly lower level of HDL-cholesterol compared with that of mice in the STZ-EPC-VLA4 group. In diabetic dyslipidemic animals the level of plasma tryglicerides was about double the value measured in dyslipidemic but non-diabetic animals, easily explainable by the altered energy metabolism known to occur in diabetes (Table 2).

In no group, significant liver toxicities were observed as indicated by ALT and AST values in the normal range and plasma creatinine indicated good renal function in all treatment animals (Table 2).

### 3.2. EPCs Reach Their Target Sites

Because organ distribution studies required PKH26 staining of injected EPCs, a different, smaller, lot of animals was assembled for this task (Table 3).

**Figure 2:**
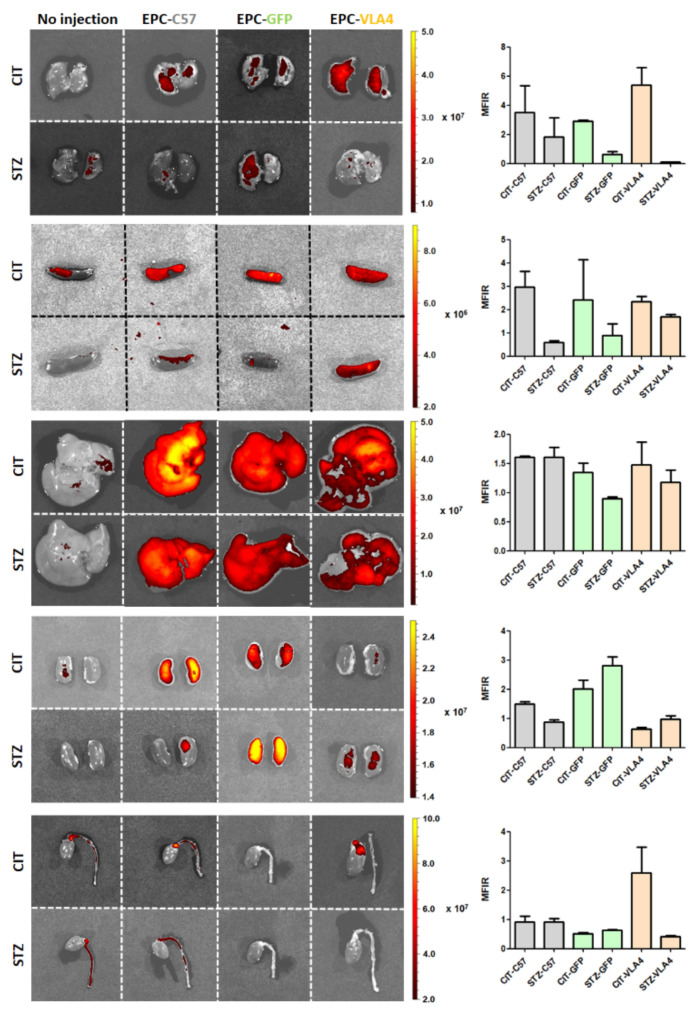
Organ distribution of EPCs. The graphs show the mean from duplicate measurements. Due to the low number of animals used (n = 2 per group), no statistical analyses were performed.

As the PKH26-EPCs were intravenously injected into the retro-orbital plexus, the possibility of a pulmonary first-pass had to be examined. In our experiments, EPCs are retained in relatively low quantities in the lungs, regardless of treatment, with a trend of lower retention in diabetic dyslipidemic mice (Figure 2), suggesting that the pulmonary first-pass effect does not significantly impact EPC-VLA4 therapy. The higher pulmonary fluorescence observed in dyslipidemic non-diabetic animals could therefore reflect a higher specific recruitment rather than a first-pass effect (Figure 2).

The overall higher splenic levels of fluorescence in dyslipidemic non-diabetic animals are in line with an increased removal of circulatory EPCs in these animals at the time point checked, perhaps as fewer cells are actively recruited in these less affected animals, while the consistent higher renal levels of fluorescence observed for the EPC-GFP is indicative of earlier removal from circulation, lysis, and excretion of PKH26 (Figure 2).

The overall low relative fluorescence levels in the aorta and heart with the sole exception of CIT-EPC-VLA4 mice could be explained by the already high auto-fluorescence in the not-injected animals masking the specific signal and does not necessarily imply lack of recruitment at these levels as other highly vascularised organs, such as the liver, show robust fluorescence increases over organ autofluorescence (Figure 2).

### 3.3. VLA4 Overexpressing EPCs Protect the Valve Function as Well as Healthy EPCs

**Figure 3:**
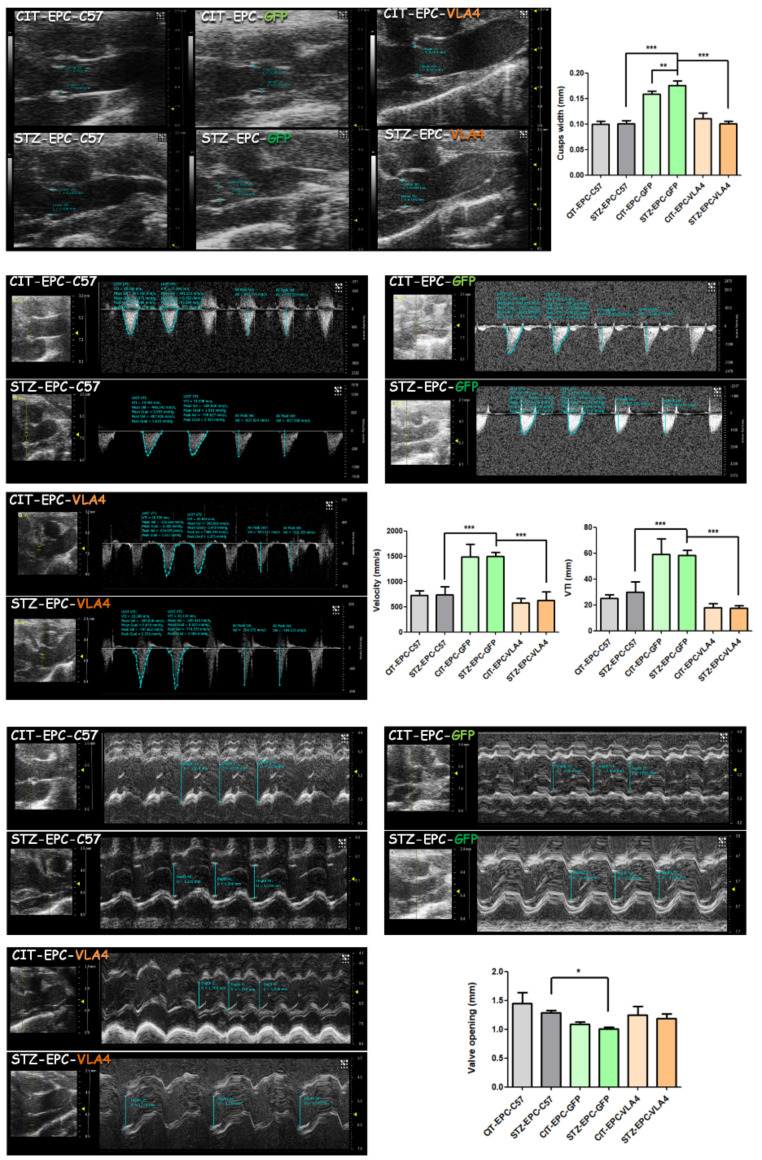
Morphologic and functional echocardiographic measurements. Shown are representative images of B-mode measurements of leaflets width and subsequential statistical analysis (**Upper panel**); pulsed-wave Doppler mode (PW Doppler-mode) measurements of the left ventricle outflow tract (LVOT), velocity-time integral (VTI) and Velocity (**Middle panel**); and M-mode measurements of maximum systolic valve opening (**Lower panel**). ANOVA, Bonferroni post-test were applied: * *p* < 0.05, ** *p* < 0.01, *** *p* < 0.001.

Echocardiographic measurements in M-mode showed a significantly reduced aortic valve opening in diabetic dyslipidemic animals treated with GFP-modified EPCs compared with that in diabetic dyslipidemic animals treated with healthy EPCs (* *p* < 0.05, Figure 3-lower panel). For diabetic dyslipidemic animals treated with diseased EPCs transfected to overexpress VLA4, aortic valve opening had similar values to those measured in healthy EPCs treated diabetic dyslipidemic animals (Figure 3-lower panel). Moreover, echocardiographic measurements taken in B-mode for aortic leaflet width revealed that diabetic dyslipidemic or dyslipidemic mice treated with GFP-modified EPCs had significantly increased leaflet widths compared to diabetic dyslipidemic or dyslipidemic mice treated with either healthy EPCs or VLA4-EPCs (*** *p* < 0.001, Figure 3-upper panel).

These morphologic findings were further sustained by the associated functional changes measured in PW Doppler-mode that showed aortic valve blood velocity (VEL) and velocity-time integral (VTI) of the left ventricle outflow tract (LVOT) were significantly higher in GFP-modified EPCs treated mice, both diabetic dyslipidemic and non-diabetic dyslipidemic, as indicative of aortic valve stenosis (Figure 3-middle panel). As can be observed in Figure 3-middle panel both treatment with healthy EPCs, as well as treatment with VLA4-modified EPCs, preserved the aortic valve function in similar degrees compared to the unfavourable evolution after treatment with GFP-transfected EPCs as negative control (*** *p* < 0.001).

### 3.4. Inflammation and Remodelling Profiles Assessed by Immunochemistry

**Figure 4:**
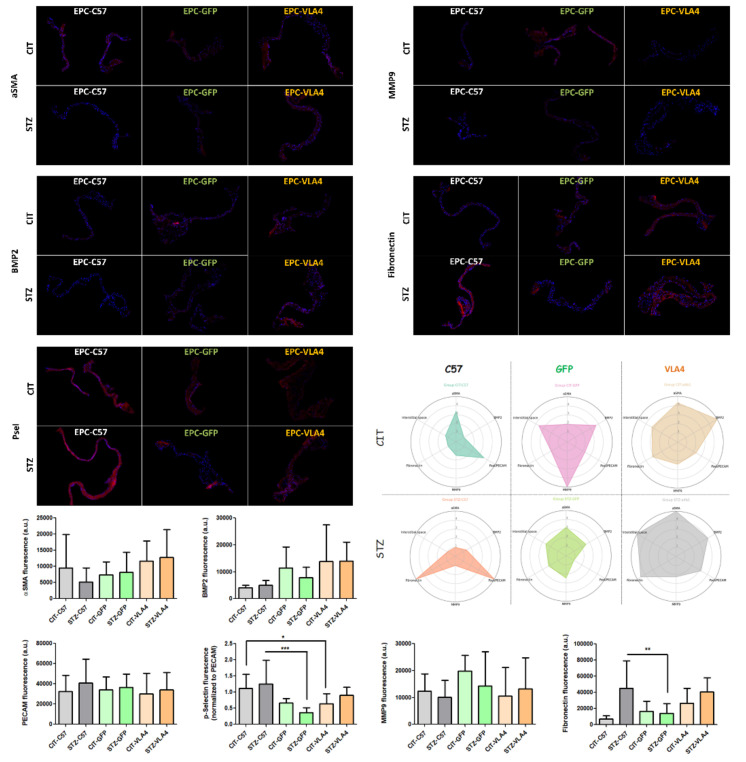
Representative immunochemistry images showing the inflammation and remodelling markers tested. The radio diagrams show the normalized profiles for each treatment condition obtained from the data shown in the bar graphs. The statistical differences were tested by Bonferroni Multiple Comparison Tests (CIT-EPC-C57 vs. CIT-EPC-GFP, CIT-EPC-C57 vs. CIT-EPC-VLA4, STZ-EPC-C57 vs. STZ-EPC-GFP and STZ-EPC-C57 vs. STZ-EPC-VLA4) and the significant results were noted as * *p* < 0.05, ** *p* < 0.01, *** *p* < 0.001.

Immunochemistry analysis of aortic valve sections showed a high level of inflammation markers Fibronectin and p-Selectin (reported normalized to the relative endothelial abundance, as p-Selectin/PECAM ratio) but relatively low remodelling markers BMP2, MMP9, αSMA and interstitial space (area/number of cells) for the animals treated with healthy EPCs (CIT-EPC-C57 and STZ-EPC-C57). In GFP-modified EPC treated mice Fibronectin and p-Selectin were reduced (STZ-C57 vs. STZ-GFP, *p* < 0.01 ** for fibronectin and *p* < 0.001 *** for p-Selectin, Bonferroni Multiple Comparison Test), however relatively high MMP9 and intermediate BMP2 levels were observed. Treatment with VLA4-modified EPC led to a mixed profile with intermediately increased inflammation markers Fibronectin and p-Selectin, high BMP2 and αSMA but low MMP9 (Figure 4).

**Figure 5:**
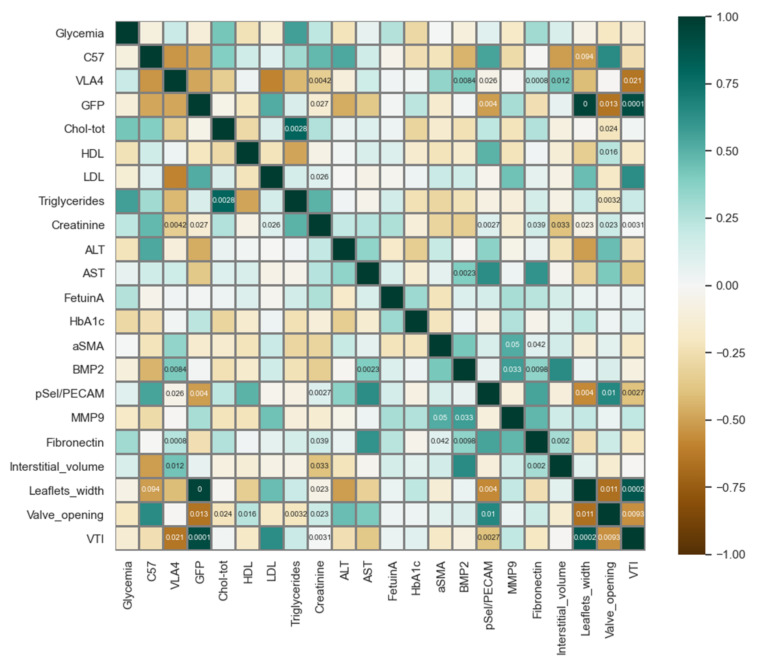
Correlations between plasma, echocardiographic and immunochemistry parameters. The colour scale codes for the Pearson coefficient value (dark green—perfect positive correlation, white—no correlation, and dark brown—perfect negative correlation). Significant correlations (*p* < 0.05) are indicated by the *p*-value noted inside the respective box. C57, VLA4 and GFP are synthetic parameters indicating the received treatment (1 if yes, 0 if no).

As an adhesion molecule, p-Selectin is involved in the recruitment of both inflammatory cells as macrophages secreting a panel of factors, such as MMP9, as well as in the recruitment of additional EPCs with roles in endothelial repairment. Among the markers studied, p-Selectin is the only marker that correlated with aortic valve function as assessed by echocardiography, with higher p-Selectin levels being associated with an overall better function (higher valve opening, *p* = 0.01, and lower LVOT VTI, *p* < 0.01, see Figure 5). MMP9 showed high correlations with the remodelling markers BMP2 and αSMA, as did fibronectin, but not p-Selectin, hinting to the sequential progression of the aortic valve pathology, with markers for inflammation and remodelling being expressed at different time points.

## 4. Discussion

Here, we show the effects of EPCs modified to overexpress VLA4 on the aortic valve function of dyslipidemic or diabetic dyslipidemic mice. The treatments were performed for a short time after the STZ-mediated destruction of pancreatic islets and atherogenic diet, as our previous experiments showed significant modifications indicative of valvulopathy only 9 days after the first STZ injection and 4 days after the diet change [8]; thus, the treatments used here are intended to delay the adverse evolution of vascular diseases and not to revert heavily diseased valves to a healthier state. As a positive control, we assayed the treatment with EPCs originating from healthy C57BL mice and diseased EPCs sham transfected with GFP was used as a negative control.

The 7-day atherogenic diet (consisting of 1% cholesterol and 15% butter supplemented standard pellets) successfully induced dyslipidemia in all animals and glycaemia levels were robustly increased in all groups with STZ-administration.

However, variability in animal response to STZ was to be expected and the mice in the STZ-EPC-VLA4 group presented faster glycaemia increases (significant increases before EPC treatment).

EPCs administered in the retro-orbital venous should transit the superior cava vein–right heart–pulmonary circulation–left heart–systemic circulation to reach their intended sites of action. To exclude the possibility that intravenously administered cells could be removed from circulation at the first-pass through the lungs and obtain information about their distribution and turnover, experiments that used PKH26-labeled cells were performed. The data showed the absence of a marked pulmonary first-pass effect, distribution in highly vascularised organs and some splenic removal from circulation, with higher renal fluorescence in the case of negative control GFP-modified EPCs hinting to earlier lysis and renal excretion of PKH26; these results show that administered EPCs reach their intended sites and mimic the physiological cycle of circulatory cells, an essential aspect for the employment in therapeutic methods.

The immunochemistry experiments performed here show different degrees and stages of inflammation and remodelling in all treatment groups with high inflammation–low remodelling for healthy EPCs–treated animals, low inflammation–high remodelling for GFP-modified EPCs and moderate inflammation–high remodelling (although low MMP9) for VLA4-modified EPCs. In our previous work using this animal model, BMP2 was increased in the aortic valves of dyslipidemic and diabetic dyslipidemic animals at 8 days after the experiments start and significantly reduced at 11 days, hinting that BMP2 is transiently increased in the early stage of valvulopathy [8]. Thus, the higher BMP2 levels observed in the experiments presented herein for the VLA4-modified EPCs treated animals (dyslipidemic or diabetic dyslipidemic mice) on the 11th day suggests a delay in the valvulopathy onset; moreover, the increase in MMP9 at 11 days we previously observed was now only present in GFP-EPCs treatment groups and not in C57-EPCs or VLA4-EPCs treated mice (dyslipidemic or diabetic dyslipidemic mice). MMP9 protein increases indicate an escape from the modulatory actions of tissue inhibitors of MMPs (TIMPs), leading to the destruction of the tissue structure, and are observed in aortic valve stenosis [28], in concordance with the functional results obtained herein.

A limitation of the study is a relatively small number of mice (n = 4) in each treatment, group allowing some baseline differences between groups (such as more STZ responsive mice in the STZ-EPC-VLA4 group); however, a larger number was hard to obtain as all experiments, including organ distribution assays and EPC isolation groups involved in total the use of 122 mice. As confounding variables for the treatment, total cholesterol and triglycerides were negatively correlated with the valve opening and positively with LVOT VTI (*p* < 0.05 and *p* < 0.01, respectively) and, such, the variability in the severity of the disease induced by diet and STZ injections makes the observed inflammation and remodelling profiles harder to interpret.

The in vivo morphologic and functional measurements performed by echocardiography show a worsening of the aortic valve function in GFP-modified EPCs treated animals (dyslipidemic or diabetic dyslipidemic mice), namely both a reduction in the valve opening and an increase in LVOT VTI. Furthermore, both healthy EPCs and VLA4-modified EPCs preserved the valve architecture and function, or at least retarded an unfavourable evolution for both diabetic dyslipidemic and non-diabetic dyslipidemic mice.

Thus, our results show that EPC transfection with α4 and β1 integrins aimed at restoring the normal VLA4 levels in diseased EPCs improves their actions, leading to an improved aortic valve function compared to EPCs transfected with a functionally irrelevant GFP protein.

The experiments described here were performed as to mimic the clinical setting in which EPCs could be derived either from the patient in need of therapy or obtained from allogenic whole blood, maybe as a byproduct of pooled plasma production, thus easing the translation to clinical applications. In the case of allogenic EPCs treatments the immunogenicity of these cells could hamper the efficacy of the treatment, however, there are reasons to believe that EPC derived EC, expressing low levels of MHC class II, have low immunogenic potential and are not attacked in the recipient organism [29]; moreover, there is an increasing volume of data showing the safety of EPC treatment, including the results presented here, as well as the observation that EPCs are, although in smaller quantities than those required for therapy, a component of the routinely administered blood transfusions.

## 5. Conclusions

Herein, EPCs were found to be important cellular tools, which after in vitro manipulation to overexpress key integrin α4β1 (also called VLA4), were injected systemically to augment aortic valve function recovery depending on their recruitment to endothelial counterparts by integrin binding. Although a role of EPCs in aortic valve therapy in diabetes and dyslipidemia has not been explicitly taken into account, the numerous studies emphasized the reduction not only of their levels in diabetes but also their functional capacity to home to the damaged endothelium, to release paracrine factors for tissue repair and subsequently to differentiate into ECs [20].

Based on our recent results that have shown that circulatory EPCs from diabetic dyslipidemic ApoE^−/−^ mice have a reduced level of VLA4, which could then contribute to poor EPC recruitment to the activated endothelium [23], we set out to investigate whether ex vivo overexpression of the α4 and β1 integrins, components of VLA4 on EPCs from diabetic or diabetic dyslipidemic ApoE^−/−^ mice, could restore part of EPC function, thereby contributing to the preservation of aortic valve function in these pathologies.

Therefore, the results of our study demonstrated that namely genetically modified allogeneic EPCs to overexpress VLA4 have the ability to preserve aortic valve function in mice with dyslipidemia and diabetes.

In conclusion, the present and future investigations on EPC function could lead to potentially novel options for therapeutic intervention in subjects with dyslipidemia and diabetes.

## 6. Patents

Process for obtaining genetically modified endothelial progenitor cells. Patent Application, OSIM No. A/00284 of 25.05.2020. Authors: Alexandru Filippi, Mihaela Loredana Antonescu (Vlad), Alina Constantin, Cristina Ana Constantinescu (Mocanu), Nicoleta Alexandru, Adriana Georgescu.

## Figures and Tables

**Table 1:** Male mice were used for biochemistry, echocardiography and immunohistochemistry determinations.

Animal Groups	Strain	Diabetic	Dyslipidemic	Injection with EPCs from C57BL or ApoE^−/−^ mice	EPCs Transfected with GFP or α_4_β_1_	Animal Number
**CIT-EPC-C57**	ApoE^−/−^	No	YES	C57BL—Healthy	-	4
**STZ-EPC-C57**	ApoE^−/−^	YES	YES	C57BL—Healthy	-	4
**CIT-EPC-GFP**	ApoE^−/−^	No	YES	ApoE^−/−^—Dyslipidemic	GFP	4
**STZ-EPC-GFP**	ApoE^−/−^	YES	YES	ApoE^−/−^—Dyslipidemic, Diabetic	GFP	4 (3)
**CIT-EPC-VLA4**	ApoE^−/−^	No	YES	ApoE^−/−^—Dyslipidemic	α_4_β_1_	4
**STZ-EPC-VLA4**	ApoE^−/−^	YES	YES	ApoE^−/−^—Dyslipidemic, Diabetic	α_4_β_1_	4

**Table 2:** Biochemistry.

	CIT-EPC-C57	STZ-EPC-C57	CIT-EPC-GFP	STZ-EPC-GFP	CIT-EPC-VLA4	STZ-EPC-VLA4	Statistical Significance ^#^
**Glycaemia (mg/dL)**	133.5 ± 17.6	235 ± 60.02	192.8 ± 23.77	280.7 ± 103.8	103.5 ± 19.71	342.5 ± 23.19	CIT-EPC-C57 vs. STZ-EPC-C57, * *p* < 0.05; STZ-EPC-C57 vs. STZ-EPC-VLA4, * *p* < 0.05; CIT-EPC-VLA4 vs. STZ-EPC-VLA4, ** *p* < 0.01
**HbA1c (%)**	3.84 ± 0.968	3.028 ± 0.565	4.164 ± 1.228	3.704 ± 0.879	4.035 ± 0.259	3.404 ± 0.338	
**Fetuin A (µg/mL)**	72.62 ± 11.28	89.54 ± 29.07	87.58 ± 36.37	76.29 ± 15.07	75.61 ± 13.63	90.53 ± 7.68	
**Triglycerides (mg/dL)**	214.8 ± 100.1	380.8 ± 55.44	158.2 ± 64.8	409 ± 132	83.84 ± 23.47	244 ± 102.7	CIT-EPC-GFP vs. STZ-EPC-GFP, * *p* < 0.05
**Total cholesterol (mg/dL)**	1224 ± 339.2	1516 ± 245.8	1124 ± 80.25	1345 ± 136	961 ± 170.9	1282 ± 106	
**HDL Cholesterol (mg/dL)**	58.18 ± 9.535	65.19 ± 7.167	68.48 ± 12.21	37.84 ± 2.119	58.87 ± 5.395	60.51 ± 11.21	CIT-EPC-GFP vs. STZ-EPC-GFP, ** *p* < 0.01; STZ-EPC-GFP vs. STZ-EPC-VLA4, * *p* < 0.05;
**LDL Cholesterol (mg/dL)**	390.4 ± 15.3	414.4 ± 9.522	449.9 ± 77.25	450.3 ± 7.774	349.4 ± 59.59	322.8 ± 110	
**ALT (U/L)**	22.1 ± 11.85	23.83 ± 6.203	8.87 ± 3.782	11.92 ± 6.915	19.39 ± 9.38	12.04 ± 2.238	
**AST (U/L)**	42.81 ± 13.21	49.15 ± 26.77	28.42 ± 6.9	36.61 ± 7.452	49.29 ± 24.67	43.01 ± 11.5	
**Creatinine (mg/dL)**	1.093 ± 0.83	1.827 ± 1.125	0.347 ± 0.463	1.396 ± 0.248	0.474 ± 0.268	0.717 ± 0.508	

*Results are given as mean ± stdev; ^#^ one way ANOVA with Bonferroni’s Multiple Comparison Test: CIT-EPC-C57 vs. STZ-EPC-C57; CIT-EPC-GFP vs. STZ-EPC-GFP; CIT-EPC-VLA4 vs. STZ-EPC-VLA4; STZ-EPC-GFP vs. STZ-EPC-VLA4; STZ-EPC-C57 vs. STZ-EPC-VLA.*

**Table 3:** Female mice were used in organ distribution assays.

Animal Groups	Strain	Diabetic	Dyslipidemic	Injection with EPCs from C57BL or ApoE^−/−^ Mice	EPCs Transfected with GFP or α_4_β_1_	Animal Number
**CIT-AFL**	ApoE^−/−^	No	YES	-	-	2
**STZ-AFL**	ApoE^−/−^	YES	YES	-	-	2
**CIT-EPC-C57**	ApoE^−/−^	No	YES	C57BL—Healthy	-	2
**STZ-EPC-C57**	ApoE^−/−^	YES	YES	C57BL—Healthy	-	2
**CIT-EPC-GFP**	ApoE^−/−^	No	YES	ApoE^−/−^—Dyslipidemic	GFP	2
**STZ-EPC-GFP**	ApoE^−/−^	YES	YES	ApoE^−/−^—Dyslipidemic, Diabetic	GFP	2
**CIT-EPC-VLA4**	ApoE^−/−^	No	YES	ApoE^−/−^—Dyslipidemic	α_4_β_1_	4
**STZ-EPC-VLA4**	ApoE^−/−^	YES	YES	ApoE^−/−^—Dyslipidemic, Diabetic	α_4_β_1_	4

## Supplementary Materials

The following supporting information can be downloaded at: https://www.mdpi.com/article/10.3390/pharmaceutics14051077/s1, Figure S1: Flowchart of the animal model establishment protocol; Figure S2: mRNA expression for the α4 and β1 integrin receptor subunits performed by RT-PCR on an aliquot of the injected EPCs (EPCs transfected with α4 and β1 integrins compared to untransfected EPCs taken as a control).
