# Supplementary figures and images for: VLA4-Enhanced Allogeneic Endothelial Progenitor Cell-Based Therapy Preserves the Aortic Valve Function in a Mouse Model of Dyslipidemia and Diabetes
Source: Pharmaceutics. 2022 May 17;14(5):1077. doi: 10.3390/pharmaceutics14051077 (PMC9143616; doi:10.3390/pharmaceutics14051077)

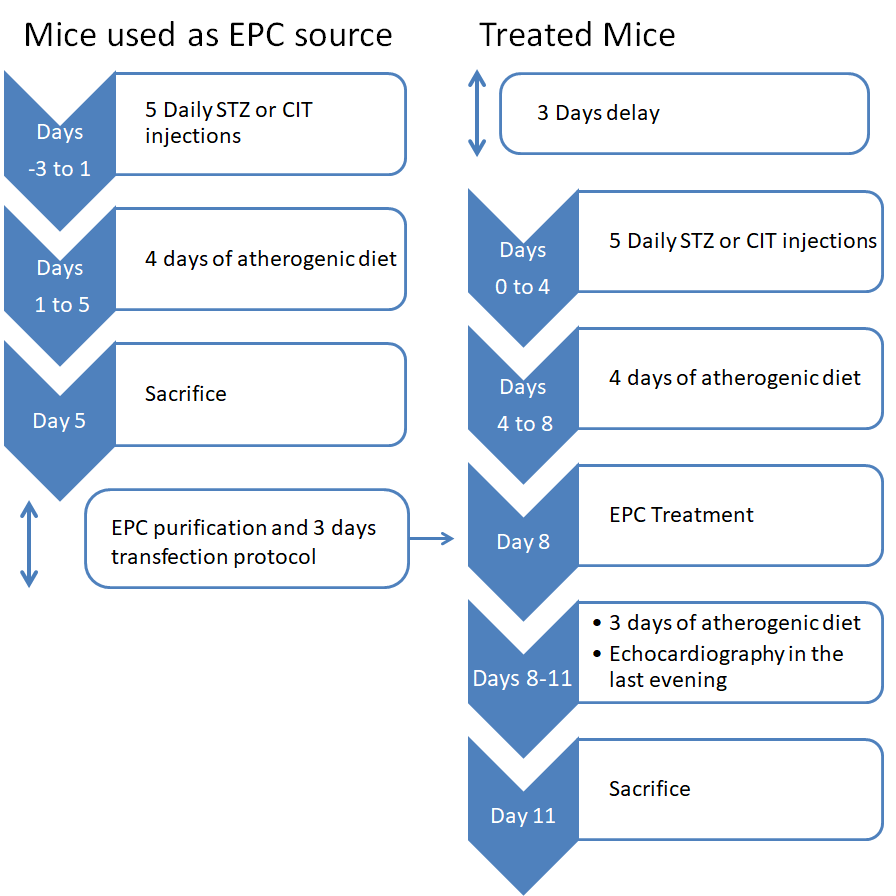

Supplement: Supplementary file 1 [file pharmaceutics-14-01077-s001.zip › Suppl-Fig1_300dpi.png]

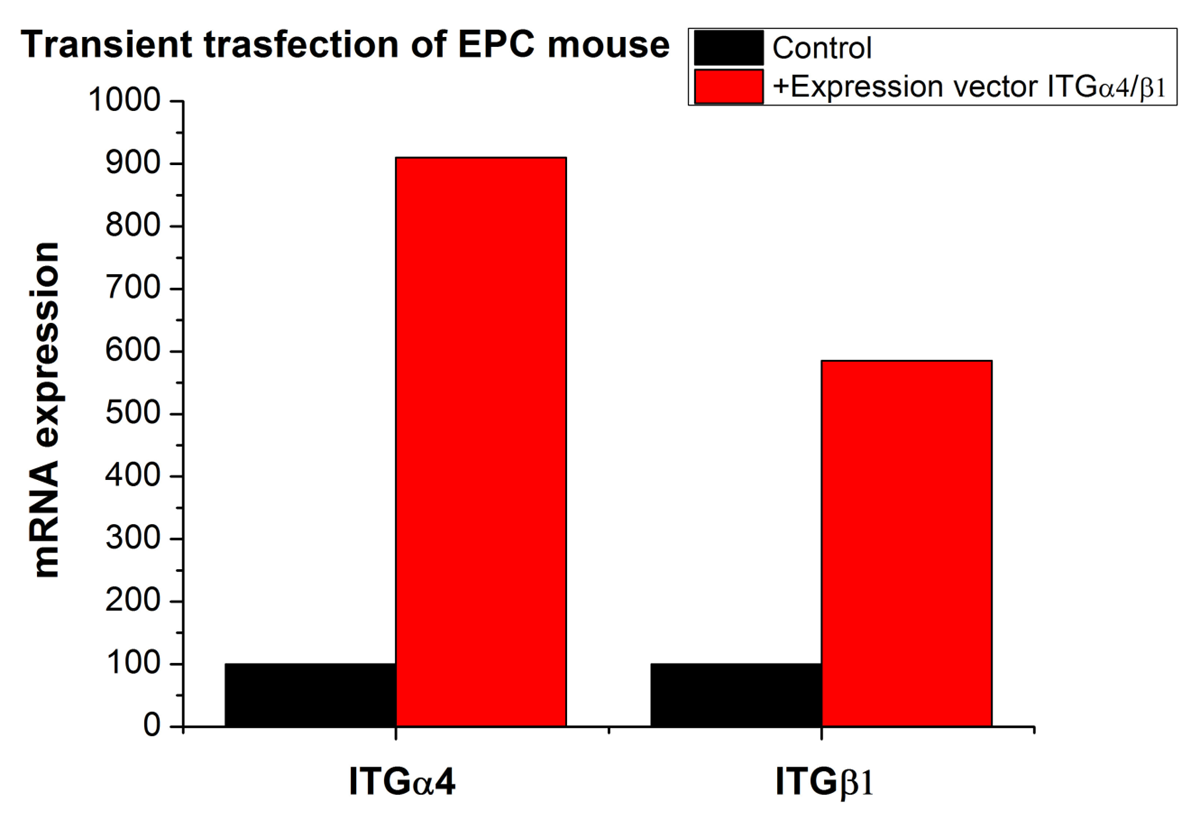

Supplement: Supplementary file 1 [file pharmaceutics-14-01077-s001.zip › Suppl-Fig2_300dpi.png]
